# Injectable-Only Overlapping Buprenorphine Starting Protocol in a Low-Threshold Setting

**DOI:** 10.1001/jamanetworkopen.2025.27016

**Published:** 2025-08-15

**Authors:** Richard C. Waters, Jeremy Hoog, Carson Bell, Penelope Toland, Joseph Valley, Lupe Hurtado, Mary Ann Kallsen, Tashay Johnson, April Gerard, Callan Elswick Fockele, Jared W. Klein

**Affiliations:** 1 Downtown Emergency Service Center, Seattle, Washington; 2 Department of Family Medicine, University of Washington School of Medicine, Seattle; 3 Department of Emergency Medicine, University of Washington School of Medicine, Seattle; 4 Department of Medicine, Division of General Internal Medicine, University of Washington School of Medicine, Seattle

## Abstract

**Importance:**

Initiating buprenorphine for opioid use disorder (OUD) in outpatient settings has become more difficult for individuals using fentanyl. Novel buprenorphine starting strategies are needed, especially for people experiencing homelessness.

**Objective:**

To evaluate the short-term outcomes of the implementation of an injectable-only overlapping buprenorphine starting protocol in a low-threshold clinic and field-based setting serving individuals with OUD and active fentanyl use.

**Design, Setting, and Participants:**

This retrospective observational cohort study was based on electronic health record data from September 2024 to January 2025 of an urban low-threshold voluntary OUD treatment program embedded in a drop-in center that offered care in the clinic and at various outreach sites, including shelters, supportive housing buildings, and encampments. Patients with moderate to severe OUD using fentanyl were included if they chose to have initial medications ordered for the injectable-only overlapping buprenorphine starting protocol between September 1 and November 30, 2024. Follow-up data were collected through January 15, 2025. Exclusion criteria were consumption of sublingual buprenorphine in the week prior, current methadone use, or lack of active fentanyl use.

**Exposure:**

The injectable-only overlapping buprenorphine starting protocol, which does not require the cessation of fentanyl use before or during the process, includes the following steps: day 1 involves a weekly 8-mg injection without preceding sublingual buprenorphine, day 2 involves a weekly 16-mg injection, and day 3 involves a monthly 128-mg or 300-mg injection.

**Main Outcomes and Measures:**

The outcomes and cascade of care were rates of protocol initiation (receipt of the initial 8-mg buprenorphine injection), completion (eventual receipt of all 3 long-acting buprenorphine injections), and 2-month retention (receipt of a second monthly buprenorphine injection within 45 days of the first).

**Results:**

Ninety-five individuals met the inclusion criteria. These patients had a median (IQR) age of 39 (23-69) years and included 52 men (55%), 75 (79%) of whom were experiencing homelessness or living in permanent supportive housing. Of the 95 patients included, 85 (90%) initiated the protocol, 71 (75%) eventually completed the protocol, and 61 (64%) received a second monthly long-acting buprenorphine injection.

**Conclusions and Relevance:**

This retrospective cohort study found that a novel injectable-only overlapping buprenorphine starting protocol had a relatively high rate of completion and 2-month retention in a low-threshold setting for individuals with OUD who used fentanyl, most of whom were experiencing homelessness. These findings suggest that this protocol is a promising potential pathway for patients to start buprenorphine in the outpatient setting.

## Key Points

**Question:**

What outcomes are associated with an injectable-only buprenorphine starting protocol in a low-threshold outpatient setting among individuals with opioid use disorder using fentanyl?

**Findings:**

In this cohort study of 95 patients with moderate to severe opioid use disorder using unregulated fentanyl, these patients chose a protocol that involved 3 escalating long-acting injectable buprenorphine doses over 3 days without requiring cessation of fentanyl and without sublingual buprenorphine. Most of these individuals (75%) completed the protocol and 64% received a second monthly long-acting injectable buprenorphine dose.

**Meaning:**

The findings suggest that an injectable-only buprenorphine starting protocol is a promising potential pathway for patients to start this lifesaving medication in the outpatient setting.

## Teaser

This cohort study describes the outcomes associated with use of an injectable-only buprenorphine initiation protocol in patients with moderate to severe opioid use disorder and active fentanyl use.

## Introduction

Unregulated fentanyl has substantially increased overdose fatalities in the community of drug users.^1^ Buprenorphine has been shown to effectively treat opioid use disorder (OUD), reducing all-cause mortality and overdose mortality^2^; however, only approximately 20% of individuals who would benefit from medication for OUD receive treatment.^3^

Initiating buprenorphine for OUD has become more challenging in the context of widespread unregulated fentanyl.^4^ Novel strategies for starting buprenorphine in acute care settings have shown promise.^5,6^ However, implementing these approaches in outpatient settings can be challenging.

Barriers to successful buprenorphine initiation include the multistep complexity of overlapping sublingual (low dose) buprenorphine starting protocols,^7^ the need to cease fentanyl use in withdrawal-first (high dose) protocols,^8^ and deliberate precipitation of withdrawal in naloxone-assisted methods.^9^ Such barriers can be heightened for people who experience homelessness, while the overdose risk among this population makes access to medications for OUD more crucial than ever.^10,11^

There is considerable interest in the role long-acting injectable buprenorphine may have in reducing overdose risk and supporting patients who aim to reduce or stop using unregulated fentanyl.^12,13^ Implementation of long-acting injectable buprenorphine has been associated with high patient satisfaction; favorable changes in abstinence, accessibility, employment, and social relationships; and reduced fentanyl use in people with OUD.^12,14,15^

In February 2025, the US Food and Drug Administration approved label changes for a monthly long-acting injectable buprenorphine product to include a rapid initiation protocol after a single 4-mg dose of sublingual buprenorphine to lessen practical obstacles to medication initiation.^16,17^ The manufacturer of the weekly long-acting injectable buprenorphine product also recommends a sublingual buprenorphine dose of at least 4 mg prior to injectable buprenorphine initiation.^18^ Recent evidence suggests that long-acting injectable buprenorphine can be effectively used to initiate patients in emergency department settings with minimal risk of precipitated withdrawal, provided these patients have a Clinical Opiate Withdrawal Scale (COWS) score of 4 or higher (with higher scores indicating greater opioid withdrawal).^19^

Achieving tolerance to 4 mg of transmucosal buprenorphine or waiting to achieve a COWS score of at least 4, however, can still present a barrier to long-acting injectable buprenorphine initiation. In response to such challenges, in routine care, we initially offered a modification of the drug label recommendations for the weekly long-acting injectable buprenorphine formulation. The drug label recommends, after reaching tolerance to 4 mg of sublingual buprenorphine, a sequence of up to 3 weekly injections administered within the first week: a weekly 16-mg injection, weekly 8-mg injection, and another weekly 8-mg injection if needed. Consolidation into a higher-dose weekly or monthly formulation is recommended at the start of the second week.^18^ Our initial modified protocol changed this sequencing. Patients were encouraged to achieve tolerance to a total daily dose of 2 to 3 mg of sublingual buprenorphine before receiving an initial 8-mg weekly injection, followed by a 16-mg weekly injection the next day, and a monthly injection thereafter. Some individuals declined any sublingual buprenorphine, due to prior negative experiences with the sublingual product, and, with informed consent, proceeded with a weekly 8-mg injection, without substantial subsequent withdrawal. Based on these experiences, we began offering an injectable-only overlapping buprenorphine starting protocol as an alternative method to individuals with moderate to severe OUD and active fentanyl use. This retrospective observational cohort study describes the short-term outcomes of the implementation of an injectable-only overlapping buprenorphine starting protocol in a low-threshold clinic and field-based setting.

## Methods

### Design and Settings

We conducted a retrospective cohort study of individuals with OUD using unregulated fentanyl who chose an injectable-only overlapping buprenorphine starting protocol between September 1 and November 30, 2024. The University of Washington Institutional Review Board approved all study procedures and waived the informed consent requirement because of the retrospective and low-risk nature of the study. We followed the Strengthening the Reporting of Observational Studies in Epidemiology (STROBE) reporting guideline.

Individuals voluntarily sought treatment at the Downtown Emergency Service Center’s Opioid Treatment Network, a low-threshold outpatient OUD care program in Seattle, Washington. Individuals served at this clinic are primarily experiencing chronic homelessness and housing instability or living in permanent supportive housing. Individuals sought care in person at the clinic or were engaged through outreach by clinic staff. Patient-labeled long-acting buprenorphine injectables were provided in the clinic or at outreach sites, including at shelters or supportive housing buildings. Nearly all patients were covered by Washington State Medicaid or were dual-eligible with Medicare due to disabling conditions. Weekly and monthly forms of long-acting injectables were paid for by Washington State Medicaid without need for prior authorization.

#### Participants

Patients with moderate to severe OUD using unregulated fentanyl were included in the study if they had a weekly long-acting buprenorphine 8-mg injection ordered and paid for by insurance between September 1 and November 30, 2024, with an intent to start the injectable-only overlapping buprenorphine starting protocol. Follow-up data were assessed through January 15, 2025. OUD was confirmed based on patient-reported history, corroborated by medical records, prior buprenorphine prescriptions, and (if needed for diagnosis) point-of-care urine toxicology testing. We excluded individuals who reported consumption of sublingual buprenorphine in the week prior to the 8-mg injection, current methadone use, lack of active fentanyl use, or choice of another buprenorphine starting protocol.

#### Protocol Description

The injectable-only overlapping buprenorphine starting protocol (Table 1) includes the following steps, without requiring the cessation of fentanyl use before or during the process: day 1 involves a weekly 8-mg injection without preceding sublingual buprenorphine; day 2 involves a weekly 16-mg injection; and day 3 involves a monthly, long-acting 128-mg or 300-mg buprenorphine injection. These 2 monthly doses were offered to provide choice and accommodate patient preferences, paired with the counseling that the 300-mg dose would likely result in higher buprenorphine levels and a larger subcutaneous depot and that the 128-mg dose would likely result in lower buprenorphine levels and a smaller subcutaneous depot.^16,18^

**Table 1.** Summary of the Injectable-Only Overlapping Buprenorphine Starting Protocol^a^

Day	Long-acting injectable buprenorphine administered	Guidance for delayed buprenorphine injections^b^
1	Weekly 8-mg injection	NA
2	Weekly 16-mg injection	Offer the weekly 16-mg injection up to 2 d after the initial 8-mg injection before restarting.
3	Monthly 128-mg or 300-mg injection	Offer the monthly injection up to 3 d after the 16-mg injection before repeating a 16-mg injection. Consider restarting the full protocol if 7 d have passed since the 16-mg injection.

Abbreviation: NA, not applicable.

^a^ See the Box for the routine patient counseling provided to patients who selected the protocol.

^b^ Decisions on restarting after delayed injections are always informed by shared decision-making and patient preferences.

All patients who chose the protocol were provided with counseling that was based on prior clinical experience (Box). Standard patient counseling included a notification that, during the starting protocol, some degree of opioid withdrawal may be likely and severe opioid withdrawal remained possible. As with sublingual-based overlapping protocols, continued unregulated full agonist opioid use was assumed. Ceasing fentanyl or other full agonist opioid use during the process may result in opioid withdrawal and prompt the need to pivot to other buprenorphine starting methods. All patients were provided naloxone and counseled on safe use strategies.

Box. **Routine Patient Counseling**

- Expect some opioid withdrawal symptoms during the 3-day process.
- Severe opioid withdrawal still remains a possibility.
- Continued use of unregulated fentanyl is assumed. Ceasing fentanyl or other full agonist opioid use during the process may result in opioid withdrawal and prompt the need to pivot to other buprenorphine starting methods.
- When using unregulated opioids, use them as safely as possible and carry naloxone.
- Supplemental sublingual buprenorphine is recommended as needed for continued fentanyl cravings, starting at 24 hours after the monthly injection.
- Use adjunct support medications provided (typically clonidine, hydroxyzine, and ondansetron) as needed for withdrawal symptoms.

If injections were not given on sequential days, the 16-mg injection was given as late as 2 days after the initial 8-mg injection before restarting. The monthly injection was given as late as 3 days after the 16-mg injection before needing to repeat a 16-mg injection. The protocol was fully restarted if a week had passed since the 16-mg injection.

Clonidine, hydroxyzine, and ondansetron were offered to treat opioid withdrawal symptoms if they occurred. Supplemental sublingual buprenorphine was provided at the time of the monthly injection, to be taken as needed for opioid cravings no earlier than 24 hours after the monthly injection.

### Clinic and Pharmacy Procedures

Individuals interested in the injectable-only overlapping protocol met with a clinician to discuss their OUD and to establish individualized treatment goals. Visits with a clinician happened either in person at the low-barrier clinic on a walk-in basis or remotely via telehealth. Telehealth visits were facilitated by a care team member who accompanied the patient at an outreach site.

All medications needed for the injectable-only overlapping protocol were prescribed to a local pharmacy and were delivered to the clinic often within 1 business day. In instances where same-day medications were needed, a licensed health care professional retrieved the medication directly from the pharmacy.

Medications were securely stored at the clinic, with a documented chain of custody for all removals and additions of controlled medications. When outreached-based care was indicated, licensed health care professionals transported and administered pharmacy-dispensed, patient-labeled medications to individuals at outreach sites, including supportive housing units, emergency housing shelters, tiny house villages, and tent encampments.

### Data Collection and Measures

All data were manually abstracted from the electronic health record (EHR) from September 2024 to January 2025, with discrepancies resolved by consensus between at least 2 authors. Demographic variables collected for this study included age, gender identity, race, and housing status. Race, gender identity, and housing status data were self-identified and documented in the EHR. Gender identity was defined as women, men, or transgender or nonbinary. Housing status was defined as unsheltered, shelter, permanent supportive housing, and non–permanent supportive housing. Race was categorized as American Indian or Alaska Native, Asian or Pacific Islander, Black, Middle Eastern or North African, White, multiracial, or unknown (ie, individuals who did not want to disclose race). Race data were collected to describe the population generalizability of individuals choosing the novel protocol. Buprenorphine injection doses and administration dates were obtained from the EHR and used to determine individuals’ progression through the injectable-only overlapping protocol.

The outcomes evaluated reflect the cascade of care. Protocol initiation rates were defined as the number of patients who received the day-1 weekly 8-mg injection divided by the total cohort who chose the injectable-only overlapping buprenorphine starting protocol as their buprenorphine initiation method. Protocol completion was defined as receiving all 3 injections in the protocol, which included those who needed a repeated weekly 8-mg or 16-mg injection to complete the process, with distinction between those who needed vs did not need any repeated weekly 8-mg or 16-mg injections to complete the 3-injection series. Two-month retention was defined as those who received a second monthly long-acting buprenorphine injection within 45 days of the first monthly long-acting buprenorphine injection. Measures of time to protocol completion were assessed, including (1) the number of days between the initial medication pharmacy order and the administration of the initial weekly 8-mg injection as well as the first monthly long-acting buprenorphine injection and (2) the number of days between the administration of the initial weekly 8-mg injection and the administration of the first monthly long-acting buprenorphine injection. Retention rates and days between injections were treated as continuous variables.

### Statistical Analysis

Descriptive statistics are reported for the cohort. The association between housing status and receipt of a second monthly buprenorphine injection—evaluated due to concerns that a 3-day injection series may be more burdensome for individuals living unhoused—was assessed using Pearson χ^2^ test and logistic regression. A 2-sided *P* < .05 was considered statistically significant. All analyses were conducted in RStudio, version 2023.12.1 (RStudio).

## Results

Ninety-five individuals met the inclusion criteria and chose to have medications ordered for the injectable-only overlapping buprenorphine starting protocol between September 1 and November 30, 2024. These patients had a median (IQR) age of 39 (23-69) years; included 42 women (44%), 52 men (55%), and 1 transgender or nonbinary individual (1%); and 6 (6%) self-identified as American Indian or Alaska Native, 5 (5%) as Asian or Pacific Islander, 13 (14%) as Black, 2 (2%) as Middle Eastern or North African, 48 (51%) as White, and 5 (5%) as multiracial, with 16 individuals (17%) having unknown race. Seventy-five patients (79%) were experiencing homelessness or living in permanent supportive housing. Patient characteristics are summarized in Table 2.

**Table 2.** Patient Characteristics

Characteristic	Patients, No. (%) (N = 95)
Age, median (IQR), y	39 (23-69)
Gender identity	
Women	42 (44)
Men	52 (55)
Transgender or nonbinary	1 (1)
Race, self-identified	
American Indian or Alaska Native	6 (6)
Asian or Pacific Islander	5 (5)
Black	13 (14)
Middle Eastern or North African	2 (2)
White	48 (51)
Multiracial	5 (5)
Unknown	16 (17)
Housing status	
Unsheltered	25 (26)
Shelter	26 (27)
PSH	24 (25)
Non-PSH	20 (21)

Abbreviation: PSH, permanent supportive housing.

Of the 95 patients who met the inclusion criteria, 85 (90%) initiated the injectable-only overlapping buprenorphine starting protocol with a weekly 8-mg injection. Seventy-one patients (75%) completed the protocol by receiving a monthly long-acting buprenorphine injection, with 58 (61%) completing the protocol with 3 injections and 13 (14%) requiring at least 1 additional weekly 8-mg or 16-mg injection due to gaps in the injection series. Of the 71 individuals who received the first monthly injection, 67 (94%) received a monthly 300-mg injection, with 4 (6%) receiving a monthly 128-mg injection. Sixty-one patients received a second monthly long-acting buprenorphine injection, for a 2-month retention rate of 64% (61 of 95) for individuals who chose the protocol and 72% (61 of 85) for those who initiated the protocol (Figure).

**Figure.**
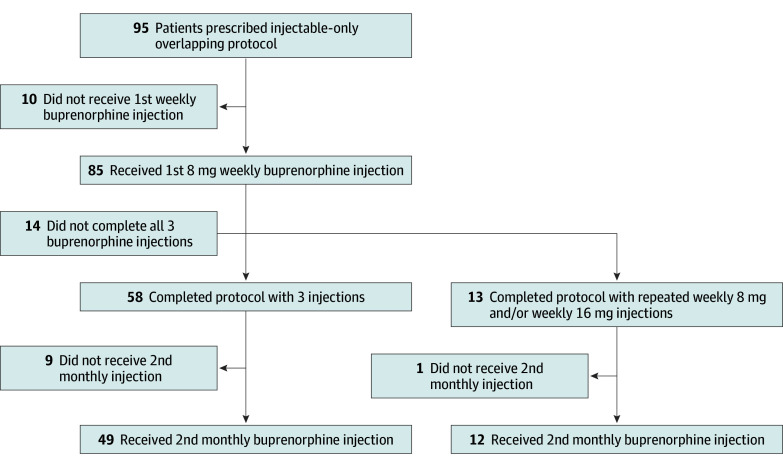
Cascade of Care for the Injectable-Only Overlapping Buprenorphine Starting Protocol

Median (IQR) times between the medication pharmacy orders and the receipt of the initial weekly 8-mg injection as well as the first monthly injection for those who received it were 4 (1-7) days and 7 (4.5-14.5) days, respectively. Median (IQR) time between the initial weekly 8-mg injection and the first monthly injection was 2 (2-3) days.

Among all 95 individuals choosing the injectable-only buprenorphine starting protocol, housing status was not associated with receipt of a second monthly injection (χ^2^_3_ = 3.67; *P* = .30). Compared with housed individuals other than those in permanent supportive housing, those living unsheltered (odds ratio [OR], 0.72; 95% CI, 0.22-2.36; *P* = .59), in temporary shelter (OR, 2.22; 95% CI, 0.63-8.31; *P* = .22), and in permanent supportive housing (OR, 1.33; 95% CI, 0.39-4.65; *P* = .65) did not have significantly different odds of progressing to a second monthly buprenorphine injection.

## Discussion

This novel injectable-only overlapping buprenorphine starting protocol is a promising alternative to existing methods for individuals, including those experiencing homelessness, with active fentanyl use who desire to start buprenorphine in an outpatient setting. With no need to stop fentanyl use before or during the protocol and using 3 serial buprenorphine injections, 64% of individuals for whom initial medications were ordered went on to receive a second monthly long-acting buprenorphine injection in this cohort.

Our findings add to the sparse literature on rates of successful completion of existing and novel buprenorphine starting protocols outside of acute care settings for individuals using fentanyl. In the largest study of overlapping sublingual buprenorphine starts (also called low-dose initiations) in outpatient settings, 35% of individuals completed the protocol, with 22% retained at 28 days.^20^ A smaller study of individuals mostly experiencing housing instability found a 37% buprenorphine retention rate at 30 days.^21^ A recent scoping review on high-dose (withdrawal-first) buprenorphine starts found only 2 case studies, with a total 9 participants, that assessed this method for individuals using fentanyl in nonfacility outpatient settings, leaving uncertainty on success rates in such settings broadly.^22^ Both overlapping sublingual (low-dose) and withdrawal-first (high-dose) starts have documented success in acute care settings, where process controls and supports are likely higher.^7,8,22^

In our cohort, 79% of individuals were experiencing homelessness or had a history of chronic homelessness (eg, those in permanent supportive housing). Prior to the emergence of widespread fentanyl, buprenorphine retention rates at 30 days for individuals experiencing housing instability were lower than the general population, with some studies reporting 37% to 45%.^23,24,25^ Use of long-acting injectable buprenorphine in those experiencing homelessness has been associated with greater buprenorphine coverage compared with sublingual buprenorphine.^26^ With a 75% eventual completion rate of this injectable-only buprenorphine starting protocol in our cohort and resulting in initiation of monthly long-acting buprenorphine injections, this starting method may be well suited for use in populations experiencing housing instability.

The injectable-only buprenorphine starting protocol builds on earlier work showing that a single weekly 24-mg dose without preceding buprenorphine given to patients with OUD in the emergency department carried a low risk (3.2%) of precipitated withdrawal for those with COWS scores of 4 to 7 and a higher risk (13.5%) for those with COWS scores of 0 to 3.^19^

Future studies should compare success rates across different buprenorphine initiation methods and identify factors in successful initiation, completion, and retention. Another area of future research is comparing variations of injectable-only protocols by using different numbers and doses of injections to assess if there are more optimal sequences of injections that maximize patient retention and minimize medication costs.

### Limitations

Our study has several limitations. Participants engaged with members of the care team and, after that engagement, requested buprenorphine medications. Thus, the injectable-only buprenorphine starting protocol may not have similar success rates with non–treatment-seeking populations. Within the course of routine clinical care, there were no standardized assessments of the patient experience after each injection. We observed that many patients experienced mild withdrawal symptoms even after the initial 8-mg weekly injection, with patient-reported severe withdrawal occurring for some, particularly after the first monthly dose. Future research should describe the patient experience and quantify the prevalence and magnitude of patient-reported opioid withdrawal symptoms experienced during this protocol. Also, while most individuals received a monthly 300-mg, rather than 128-mg, dose, this selection may have been biased by greater patient familiarity with the brand name of the 300-mg dose and greater care team experience with using this product.

Several factors limit the generalizability of this study. The program that implemented this protocol provides pharmacy-dispensed, patient-labeled long-acting buprenorphine injections at a drop-in clinic and through field-based outreach, reaching shelters, encampments, and permanent supportive housing buildings. The low-threshold clinic and outreach-based care likely contributed to the high initiation and completion rates.^27^ However, it remains unclear whether completion rates would be as high without the outreach-based component. Research assessing retention rates associated with implementation of this protocol within clinic-only settings, without an outreach component, would assist in clarifying the generalizability of this protocol to such settings. Furthermore, in our setting, the weekly and monthly injections were paid for by Medicaid insurance without prior authorization. Implementation will likely be more difficult in settings with greater logistical or financial barriers to medication access.

## Conclusions

In this retrospective cohort study of patients with moderate to severe OUD who used fentanyl and had high rates of homelessness, those who had medications ordered for an injectable-only overlapping buprenorphine starting protocol had a 75% eventual completion rate and 64% rate of receipt of the second monthly injection. These findings suggest that this protocol is a promising potential pathway for patients to start this lifesaving medication in the outpatient setting.

## Notes

Supplement.
Data Sharing Statement
