# Supplementary material for: Injectable-Only Overlapping Buprenorphine Starting Protocol in a Low-Threshold Setting
Source: JAMA Netw Open. 2025 Aug 15;8(8):e2527016. doi: 10.1001/jamanetworkopen.2025.27016 (PMC12357186; doi:10.1001/jamanetworkopen.2025.27016)
Supplement: Supplement. — Data Sharing Statement [file jamanetwopen-e2527016-s001.pdf]

Data Sharing Statement

Waters. Injectable-Only Overlapping Buprenorphine Starting Protocol in a Low-Threshold Setting. *JAMA Netw Open*. Published August 15, 2025.
doi:10.1001/jamanetworkopen.2025.27016

Data
Data available: No

Additional Information
Explanation for why data not available: We can share de-identified data upon request
